# Supplementary material for: Dairy farmer practices and attitudes relating to pasture-based and indoor production systems in Scotland
Source: PLoS One. 2022 Feb 3;17(2):e0262268. doi: 10.1371/journal.pone.0262268 (PMC8812888; doi:10.1371/journal.pone.0262268)
Supplement: S1 Text — (DOCX) [file pone.0262268.s001.docx]

**
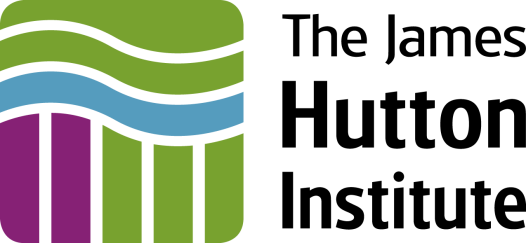
**

**Cows eat grass, don’t they? UK farmer survey**

This survey explores the views of dairy farmers in the UK and Ireland about the future of their industry.

This survey is part of a larger 3 year project on stakeholder, farmer and public views on indoor and pasture based systems in the UK and Ireland called 'Cows eat grass, don't they?' funded by the British Academy, a body that funds arts and social sciences research: [**www.docowseatgrass.org**](http://www.docowseatgrass.org/).

There has been public and media attention on dairy systems in recent years, e.g. rapid dairy expansion in Ireland, total confinement systems in the UK. **But little is known about farmers' views about the future of their industry.**

The survey is for any commercial dairy farmers in the UK and Ireland. **It takes 10-15 minutes to complete.** Questions cover production systems and opinions. If you would prefer to fill the survey in online you can go to: [**http://surveys.hutton.ac.uk/docowseatgrass**](http://surveys.hutton.ac.uk/docowseatgrass)

**As a gesture of goodwill we will donate £2 for every survey completed to charities helping struggling farmers**: the Royal Agricultural Benevolent Institution (RABI) in England and Wales; the Royal Scottish Agricultural Benevolent Institution (RSABI); Rural Support in Northern Ireland; and the Mind our Farm Families phoneline in Ireland. We aim to raise £1500 for these charities in total.

Data will be stored anonymously and will be entirely confidential. The results will be reported in a blog online, academic papers, and presentations.

If you have any questions about the survey do not hesitate to get in touch with the researcher Orla Shortall, [orla.shortall@hutton.ac.uk](mailto:orla.shortall@hutton.ac.uk) 0044 (0)1224395302 or Lee-Ann Sutherland, [lee-ann.sutherland@hutton.ac.uk](mailto:lee-ann.sutherland@hutton.ac.uk), 0044 (0)3449285428.

Thank you for your participation.

**Section 1 Farmer details**

1. **How long have you been farming?**

Under 10 years 🞏 10-20 years 🞏 20-30 years 🞏 Over 30 years 🞏

1. **What is your gender?**

Male 🞏 Female 🞏 Other 🞏

1. **What is your highest qualification?**

GCSE equivalent 🞏 A level equivalent 🞏 Certificate 🞏 Diploma 🞏 Bachelor’s degree 🞏 Postgraduate degree 🞏

1. **Are you the business:**

Owner 🞏 manager 🞏 employee 🞏 other ___________

**Section 2 Farm details**

1. **Where is your farm?**Scotland 🞏 Wales 🞏 Northern Ireland 🞏 North West England 🞏
   North East England 🞏 Midlands 🞏 South West England 🞏
   South East England 🞏
2. **Is your farm:**

Conventional 🞏 Organic 🞏

1. **How many cows do you milk?___**
2. **In relation to your dairy business how many hectares do you:**
3. Own___
4. Rent___
5. Operate under any other arrangement e.g. farming partnership___
6. **What is your average milk yield per cow per year?** ______ litres **OR** _____kg
7. **How many full time labour units operate on your farm?** _____

**Section 3 Management system**

1. **Do you operate:**
2. Year round calving 🞏
3. Block spring calving 🞏
4. Block autumn calving 🞏
5. Block spring and autumn calving 🞏
6. Other________________________________________________________
7. **Why do you operate this system?** (Please tick all that apply)
8. Because of my milk contract 🞏
9. For lifestyle reasons 🞏
10. Because of labour availability 🞏
11. For welfare reasons 🞏
12. Because it suits my facilities 🞏
13. Other (please specify) _____________________________________________
    ________________________________________________________________________________________________________________________________
14. **Which of these best describes your management system:**

Grazing

1. All year round grazing. 🞏

Grazing and housing

1. Summer grazing with minimal indoor feeding, winter housing. 🞏
2. Summer grazing with additional indoor feeding, winter housing. 🞏

Indoor

1. Some lactating cows are housed all year round. 🞏
2. All lactating cows are housed all year round. 🞏
3. All cows (including followers) are housed all year round. 🞏

**If answer d), e) or f) in question 13**

**Why did you decide to move cows inside all year round?**
(Please rank in order of importance)

1. To increase yields through feeding higher energy feed___
2. Because it was possible through the purchase of a robotic milking system___
3. Wanted to expand but couldn’t acquire more land___
4. Too far for cows to walk to parlour___
5. To reduce labour___
6. For disease control reasons___
7. Other (please specify) _____________________________________________
   ________________________________________________________________________________________________________________________________
8. **Do you use a zero grazing system to bring grass to the cows indoors?**Yes 🞏 No 🞏
9. **How old are your housing facilities? (please tick all that apply)**

0-5 years 🞏 5-15 years 🞏 15-25 years 🞏 25 years + 🞏

1. **Have your expanded milk production since 2015?**Yes 🞏 No 🞏

**If yes, how did you expand production? (Please tick all that apply)**

1. Acquired more land 🞏
2. Increased cow numbers 🞏
3. Fed more concentrate to increase yields 🞏
4. Changed cattle breeds 🞏
5. Changed calving pattern 🞏
6. Changed grass management 🞏
7. Improved health and/or fertility 🞏
8. Entered into partnership with another farmer 🞏
9. Other (please specify) _____________________________________________ ________________________________________________________________________________________________________________________________________
10. **Do you plan on expanding milk production in the near future?**Yes 🞏 No 🞏

**If yes, how do you plan to expand production? (Please tick all that apply)**

1. Acquiring more land 🞏
2. Increasing cow numbers 🞏
3. Feeding more concentrate to increase yields 🞏
4. Changing cattle breeds 🞏
5. Changing calving pattern 🞏
6. Changing grass management 🞏
7. Improving health and/or fertility 🞏
8. Entering into partnership with another farmer 🞏

**Section 3 Attitudes to farm systems**

**‘Indoor dairy farming’** is taken to mean a system where some or all cows are housed all year around. A **‘pasture based system’** is taken to mean a system where cows are grazed for at least part of the year.

1. **Cows should have access to pasture for at least part of the year.**

Strongly agree 🞏 Agree 🞏 Neither agree nor disagree 🞏
Disagree 🞏 Strongly disagree 🞏

1. **Animal welfare is better if the cows have access to pasture for part of the year.**

Strongly agree 🞏 Agree 🞏 Neither agree nor disagree 🞏
Disagree 🞏 Strongly disagree 🞏

1. **Animal welfare is better if the cows are housed all year**.

Strongly agree 🞏 Agree 🞏 Neither agree nor disagree 🞏
Disagree 🞏 Strongly disagree 🞏

1. **The farmer’s stock keeping skills are more important than the type of system (indoor or pasture based) for animal welfare outcomes.**

Strongly agree 🞏 Agree 🞏 Neither agree nor disagree 🞏
Disagree 🞏 Strongly disagree 🞏

1. **Indoor dairy farms are better for the environment compared to a pasture based system.**

Strongly agree 🞏 Agree 🞏 Neither agree nor disagree 🞏
Disagree 🞏 Strongly disagree 🞏

1. **It is easier to turn a profit on a pasture based farm.**

Strongly agree 🞏 Agree 🞏 Neither agree nor disagree 🞏
Disagree 🞏 Strongly disagree 🞏

1. **It is easier to turn a profit on an indoor farm.**

Strongly agree 🞏 Agree 🞏 Neither agree nor disagree 🞏
Disagree 🞏 Strongly disagree 🞏

1. **Neither system is more profitable but profitability depends on management.**

Strongly agree 🞏 Agree 🞏 Neither agree nor disagree 🞏
Disagree 🞏 Strongly disagree 🞏

1. **Block calving has advantages over year round calving for UK farmers.**

Strongly agree 🞏 Agree 🞏 Neither agree nor disagree 🞏
Disagree 🞏 Strongly disagree 🞏

**If agree or strongly agree**

**What are the advantages of block calving? (Please tick all that apply)**

1. Simpler 🞏
2. Lower cost 🞏
3. Better for welfare 🞏
4. Better for farmer’s lifestyle 🞏
5. Less labour 🞏
6. Lower risk 🞏
7. Other (please specify) _____________________________________________
   ________________________________________________________________________________________________________________________________
8. **Neither system (block calving or year round calving) is 'better' but the success of the farm depends on management.**

Strongly agree 🞏 Agree 🞏 Neither agree nor disagree 🞏
Disagree 🞏 Strongly disagree 🞏

1. **How satisfied are you with how profitable your dairy farm is?**

Very satisfied 🞏 Satisfied 🞏 Neither satisfied nor dissatisfied 🞏 Dissatisfied 🞏 Very dissatisfied 🞏

1. **How satisfied are you with your work/life balance?**

Very satisfied 🞏 Satisfied 🞏 Neither satisfied nor dissatisfied 🞏 Dissatisfied 🞏 Very dissatisfied 🞏

1. **What do you see as the main challenges facing the dairy sector in the UK? (please rank in order of importance)**
2. Low milk prices___
3. High costs___
4. Overwork for farmers___
5. Lack of adequate government support___
6. Change in consumer diets away from dairy produce___
7. Bad/variable weather conditions___
8. Lack of labour availability__
9. Other (please specify) _____________________________________________
10. **Which metrics do you find the most helpful when benchmarking your farm/assessing the productivity of your farm?
    (please tick all that you use)**

**Milk production**

1. Milk production (litres) per hectare per year 🞏
2. Milk production (litres) per cow per year 🞏
3. Milk production (litres) per concentrate feed per year 🞏
4. Milk production (litres) per cow over their lifetime 🞏
5. Milk solids per hectare per year 🞏
6. Milk solids per cow per year 🞏
7. Milk solids per concentrate feed per year 🞏
8. Milk solids per cow over their lifetime 🞏

**Fertility and replacements**

1. Herd replacement rate 🞏
2. Pregnancy rate 🞏
3. Age at first calving 🞏
4. Cows calved within first 6 weeks of block calving 🞏
5. Other (please specify)______________________________________
6. Other (please specify)______________________________________
7. **Do you have any additional comments about this topic?** ________________________________________________________________________________________________________________________________________

**Would you like receive information about the results of the study?
Yes** 🞏 **No** 🞏

**If yes, please enter your details according to how you would like to be contacted.**

**Your data will be stored confidentially and will not be linked to your survey responses.**

Name________________

Email address___________________

Phone number ______________________

**The next stage of the research involves one to one interviews with farmers to explore their decision making about their own farm and their views on the future of the dairy sector in more detail.**

**Interviews usually last about an hour and take place in person, at a place convenient for the interviewee, or by phone.**

**Would you like to take part in an interview?
Yes** 🞏 **No** 🞏

**If yes, Please enter your details according to how you would like to be contacted.**

**Your data will be stored confidentially and will not be linked to your survey responses.**

Name________________

Email address___________________

Phone number ______________________

**Thank you for your participation.**
As a gesture of goodwill we will donate £2 for every survey completed to charities helping struggling farmers: the Royal Agricultural Benevolent Institution (RABI) in England and Wales; the Royal Scottish Agricultural Benevolent Institution (RSABI); Rural Support in Northern Ireland; and the 'Mind our Farm Families' phoneline run by Pieta House and the IFA in the Republic of Ireland.
