# Supplementary material for: Dairy farmer practices and attitudes relating to pasture-based and indoor production systems in Scotland
Source: PLoS One. 2022 Feb 3;17(2):e0262268. doi: 10.1371/journal.pone.0262268 (PMC8812888; doi:10.1371/journal.pone.0262268)
Supplement: Attachment — Submitted filename: Comments to the Authors.docx [file pone.0262268.s003.docx]

Comments to “Dairy farmer practices and attitudes relating to pasture-based, high- input and indoor production systems in the UK and Ireland”.

This paper addresses a current topic with a keen approach. The issue that is questioning livestock in the most acute way, its carbon footprint, is only slightly addressed; however, I have no doubt that these results will become one of the guidelines for a very up-to-date discussion in many countries.

Line 18: and many others following below; lack of consistency, in some lines “year round” is used and in others “year-round”.

Line 30: should be “Scotland,”

Lines 57 to 63: this contradiction should not be left unsolved (lines 57 -60) “Year-round housing allows farms to … have greater control over the health … the cows; and (line 62) “Evidence has suggested that indoor dairy farming can result in worse health outcomes for cows”.

Lines 64 and 65: “cows’ emotional wellbeing” is more accurate than “cows’ subjective wellbeing”.

Lines 67 and 68: “many call for more up to date research and greater understanding of the implications of year-round housing for animal health and welfare” Leaving this assessment unchallenged apparently ignores findings in the last few years based on solid research demonstrating the benefits to animal welfare from the access to pasture (*e.g. Arnott G., Ferris C. P. &O’Connell N. E. (2017). Review: welfare of dairy cows in continuously housed and pasture-based production systems. Animal (2017), 11, 2: 261–273 doi:10.1017/S1751731116001336. Burow E., Rousing T., Thomsen P. T., Otten N. D. & Sørensen J. T. (2013). Effect of grazing on the cow welfare of dairy herds evaluated by a multidimensional welfare index. Animal 7:5, pp 834–842 doi:10.1017/S1751731112002297 Mee J. F. & Boyle L. A. (2020). Assessing whether dairy cow welfare is “better” in pasture-based than in confinement-based management systems. New Zealand Veterinary Journal, 68,3: 168-177.)*

Lines 74 to 77: greenhouse gas emissions from the agricultural and transport systems involved in making feedstuffs available to housed cattle should not be excluded from such estimates, which otherwise are biased.

Line 156-157: I appraise that the answer of the authors given to Reviewer #2 on this issue is uncomplete, since there are well accepted methods used to remove outliers.

Line 289: “farm is. 35% of” should be “farm is; 35% of”

Line 328: “they are operate” should be “they operate”.

Line 362: should be “Gonzalez-Mejia”

Line 430: should be “in biophysical”; should be of spring.
