# Supplementary material for: Dairy farmer practices and attitudes relating to pasture-based and indoor production systems in Scotland
Source: PLoS One. 2022 Feb 3;17(2):e0262268. doi: 10.1371/journal.pone.0262268 (PMC8812888; doi:10.1371/journal.pone.0262268)
Supplement: Attachment — Submitted filename: Reviewer comments PlosOne.docx [file pone.0262268.s002.docx]

We have responded to the editor and reviewers’ comments in blue below.

PONE-D-19-31729
Dairy farmer practices and attitudes relating to pasture-based, high-input and indoor production systems in the UK and Ireland
PLOS ONE

Dear Dr Shortall,

Thank you for submitting your manuscript to PLOS ONE. After careful consideration, we have decided that your manuscript does not meet our criteria for publication and must therefore be rejected.

Specifically:
My decision of rejecting the manuscript is based on the critical point of the diverse methodology (see specific comments below) that limits the ability to draw inferential comparisions.

In order to address this point, we separated the data from different countries and analysed only the Scottish data. The Scottish data was collected by posting the survey to all Scottish dairy farmers and making the survey available online. There is now no comparison in the paper between different countries and no divergent data collection methods.

I have to apologize for the long duration of this review process, it was nearly impossible to find reviewers, which is an increasing problem throughout the community.

I am sorry that we cannot be more positive on this occasion, but hope that you appreciate the reasons for this decision.

Yours sincerely,

Edna Hillmann, Ph.D.
Academic Editor
PLOS ONE


[Note: HTML markup is below. Please do not edit.]

Reviewers' comments:

Reviewer's Responses to Questions

**Comments to the Author**

1. Is the manuscript technically sound, and do the data support the conclusions?

The manuscript must describe a technically sound piece of scientific research with data that supports the conclusions. Experiments must have been conducted rigorously, with appropriate controls, replication, and sample sizes. The conclusions must be drawn appropriately based on the data presented.

Reviewer #1: Yes

Reviewer #2: No

2. Has the statistical analysis been performed appropriately and rigorously?

Reviewer #1: Yes

Reviewer #2: Yes

3. Have the authors made all data underlying the findings in their manuscript fully available?

The [PLOS Data policy](https://protect-eu.mimecast.com/s/KswbC76NOIAVrPlf80_gP?domain=plosone.org) requires authors to make all data underlying the findings described in their manuscript fully available without restriction, with rare exception (please refer to the Data Availability Statement in the manuscript PDF file). The data should be provided as part of the manuscript or its supporting information, or deposited to a public repository. For example, in addition to summary statistics, the data points behind means, medians and variance measures should be available. If there are restrictions on publicly sharing data—e.g. participant privacy or use of data from a third party—those must be specified.

Reviewer #1: Yes

Reviewer #2: No

 We are in the process of uploading the data to an open access repository. The process will be completed shortly.

4. Is the manuscript presented in an intelligible fashion and written in standard English?

PLOS ONE does not copyedit accepted manuscripts, so the language in submitted articles must be clear, correct, and unambiguous. Any typographical or grammatical errors should be corrected at revision, so please note any specific errors here.

Reviewer #1: Yes

Reviewer #2: Yes

5. Review Comments to the Author

Please use the space provided to explain your answers to the questions above. You may also include additional comments for the author, including concerns about dual publication, research ethics, or publication ethics. (Please upload your review as an attachment if it exceeds 20,000 characters)

Reviewer #1: Comments for: Dairy farmer practices and attitudes relating to pasture-based, high-input and indoor production systems in the UK and Ireland.

This is a timely and well-written article. Its findings make a significant contribution to the understanding of the nuances within the UK and Irish dairy industries and the factors impacting farmers’ attitudes to different farming systems. The method used is well presented and explained and the findings thoroughly discussed and well-argued using contemporary literature. I recommend this article for publication subject to some of the minor comments and suggestions made below.

We’d like to thank reviewer 1 for the positive comments and we feel that we have retained these elements of the paper in the current version.

Line 24: could any key findings on the UK perspective be added in? Line 24 onwards presents some of the nuances of the Irish perspective but nothing on the UK perspective: makes it appear as though only part of the story is being told here.
This is no longer relevant.

Line 19: ‘the cows’ reads somewhat awkwardly – consider changing.
This is no longer relevant.

Line 22: I found it difficult to grasp the exact meaning of this sentence, it seems somewhat unfinished – is the point that there is diversity amongst farmers regarding their preferences for indoor dairy farming so the reality is not as clear cut as saying that consumers don’t like cows being housed indoors and farmers do? Suggest rewriting this sentence to make this point clearer and more obvious (as it is an interesting and critical finding).
This is no longer relevant.

Line 35: Spelling error – should be Saudi Arabia
This is no longer relevant.

Line 36: Not quite clear what ‘consolidation’ means in this context? – the strengthening of the UK sector through increased intensification?
Consolidation means fewer, larger herds. This was clarified on lines 33-34.

Line 49: Edit reference to overall style.
References are in journal preferred Vancouver style.

Line 95: Spelling error – understanding.
Removed

Line 121: Suggest putting in brackets and changing to “(including reason for choosing calving system in the UK questionnaire)” – may help the sentence flow better.
Removed

Line 130: Can you give an example of what these different attitudinal questions are?
More details about the attitudinal questions is on line 141 and the attitudinal questions are shown in Fig2.

Line 149: Suggest including a comma after ‘Initially”.
Removed

Line 155 to 159: Had to read this sentence several times, suggest amending to make it easier to follow. Potential issue is that ‘Certain’ as the start of the sentence does not seem to fit with “that were of interest”. Also, in line 157, ‘have’ may be missing and ‘they views’ should be ‘the views’.
Removed

Line 170: Referencing error.
Removed

Line 191: As it is an abbreviation, Fig. should have a full stop after it.
Full stops were added after every use of ‘Fig’.

Line 204: Check the figure of 19% - based on the information presented in table 1, this should potentially be 20%.
Removed

Line 233: Suggest changing to “who strongly agreed”.
Removed

Line 257: Suggest adding a comma after “likely to agree”.
Removed

Line 279: remove ‘they’.
Removed

Line 279: Change of style here, writing “Fifty nine” when previous sentences use numerical style. Suggest amending to be consistent.
Removed

Line 280: Same comment as above regarding “twenty eight”.
Removed

Line 299: Requires space after “Table 4.”
Removed

Line 324: Again, consistency in how numerical information is written.
The paper was checked to for consistency in relation to this.

Line 325: Should be ‘compared’.
Removed

Line 336: Interesting finding – I wonder why this was? Do those with more cows also have more employees and could this contribute to greater satisfaction with work-life balance?
The results of this model were different for the Scottish data.

Line 394: Suggest amending attitudes to attitude.
Removed

Line 489: Interesting – indication of social constructionism at play perhaps?
Could the reviewer expand on what is meant by social constructionism here?

Line 496: Referencing error.
Removed

Line 508: This answers my earlier speculation regarding Line 336 above – great to have this in the discussion.
Removed because this result was different for the Scottish data.

Reviewer #2: This is an interesting paper, and I think that the results will be helpful to the UK and industry.
Thank you for the positive feedback. We believe that the paper based on the Scottish detail will be helpful to the dairy industry.

That said, the methodology is a bit too diverse (including different surveys, sent to different countries, using different recruitment strategies and even formats). All this may not be a problem for reporting back to the industry, but it limits the ability to draw inferential comparisions, and to group different types of participants (such as those that are part of the ‘UK’ sample).
We acknowledged this point and in order to address it, we separated the data from different countries and analysed only the Scottish data. The Scottish data was collected by posting the survey to all Scottish dairy farmers and making the survey available online. There is now no comparison in the paper between different countries and no divergent data collection methods. So we believe this has resolved the methodological issue.

I have provided some detailed comments below that I hope will be helpful to the authors.

line 17: please provide response rates?
We provide a response rate on line 17 of the abstract.

line 44-47: here or elsewhere it is important to distinguish between the environmental concern of where cows get their nutrients, and the welfare concern of whether they have access to the outdoors. The current discussion in confounded.
These lines were intended to provide a definition of terminology rather than explain the environmental and animal welfare concerns. They have been removed in this version of the paper.

line 60: I’d drop the claim about health - no evident to support this.
We cite published research to support this.

line 66: of course the calls are disputed by industry, but is this an academic argument supported by evidence? If not, I’d drop this or at least make it clear.
We would like to keep this in, as the introduction is intended to provide the reader with a background not only on the academic literature, but on media, charity and industry perspectives on these issues.

line 87: exactly - this is at the heart of the welfare vs. economic/environmental conflict.
line 106: here and elsewhere be careful with your wording on this point. The Irish system is grass based. This means that cows are outside whether they like it or not. Providing ‘access’ implies that animals have some choice in the matter, for example, that they can can go inside a barn to escape inclement weather.
This is a useful point, thank you for highlighting it. The question in the Scottish survey about grazing was worded in terms of ‘access’. We acknowledge this isn’t the best wording, based on your point. We think it doesn’t undermine the results because there are very few farms in Scotland where cows could go outside or inside at will, so it’s unlikely respondents interpreted it in this way. Though we agree wording it along the lines of ‘cows should graze for part of the year’ would’ve been a better choice of words.

line 107: don’t conflate the economic claims with the system.
Removed

line 116: does separate mean different? Might differences in what was asked lead to differences in the responses? Can these be made available as ESM?
This part was removed, but we made the survey available as supplementary material.

line 139: Might responses differ for the different methods (paper vs. online?)
Removed

line 147: These needs more explanation - ‘considered’ on the basis of what criteria?
The data that were removed were anomalous number entries, for instance if someone entered ’90,000’ litres per cow per year, we assumed that was a mistake and deleted it.

line 227 and elsewhere: given that you conducted surveys separately in Scotland, Northern Ireland, etc., does it make sense to group as UK?
Removed

line 265: is this one of the 5 quotes - how was this selected?
We removed the inclusion of comments because we didn’t feel it added to the findings of this version of the paper.

line 377: sample size is not the same as representative. Don’t confuse the two.
Removed

line 389: you cannot make inferences about the response rate in countries other than Scotland.
Removed

6. PLOS authors have the option to publish the peer review history of their article ([what does this mean?](https://protect-eu.mimecast.com/s/rPu2C8qPOU6PmJ1T1-HUr?domain=journals.plos.org)). If published, this will include your full peer review and any attached files.


If you choose “no”, your identity will remain anonymous but your review may still be made public.


**Do you want your identity to be public for this peer review?** For information about this choice, including consent withdrawal, please see our [Privacy Policy](https://protect-eu.mimecast.com/s/pn4XC98ROFkNJZQi3f_Kq?domain=plos.org).

Reviewer #1: No

Reviewer #2: No
